# Identification of Immunogenic Proteins of *Waddlia chondrophila*

**DOI:** 10.1371/journal.pone.0028605

**Published:** 2012-01-04

**Authors:** Carole Kebbi-Beghdadi, Julia Lienard, Frederic Uyttebroeck, David Baud, Beat M. Riederer, Gilbert Greub

**Affiliations:** 1 Center for Research on Intracellular Bacteria (CRIB), Institute of Microbiology, University Hospital Center and University of Lausanne, Lausanne, Switzerland; 2 Department of Cellular Biology and Morphology, University of Lausanne, Lausanne, Switzerland; 3 Proteomics Unit, Department of Psychiatric Neurosciences, Cery, Prilly-Lausanne, Switzerland; University Freiburg, Germany

## Abstract

Evidence is growing for a role of *Waddlia chondrophila* as an agent of adverse pregnancy outcomes in both humans and ruminants. This emerging pathogen, member of the order *Chlamydiales*, is also implicated in bronchiolitis and lower respiratory tract infections. Until now, the serological diagnosis of *W. chondrophila* infection has mainly relied on manually intensive tests including micro-immunofluorescence and Western blotting. Thus, there is an urgent need to establish reliable high throughput serological assays. Using a combined genomic and proteomic approach, we detected 57 immunogenic proteins of *W. chondrophila*, of which 17 were analysed by mass spectrometry. Two novel hypothetical proteins, Wim3 and Wim4, were expressed as recombinant proteins in *Escherichia coli*, purified and used as antigens in an ELISA test. Both proteins were recognized by sera of rabbits immunized with *W. chondrophila* as well as by human *W. chondrophila* positive sera but not by rabbit pre-immune sera nor human *W. chondrophila* negative sera. These results demonstrated that the approach chosen is suitable to identify immunogenic proteins that can be used to develop a serological test. This latter will be a valuable tool to further clarify the pathogenic potential of *W. chondrophila*.

## Introduction

*Waddlia chondrophila* is an emerging pathogen belonging to the *Chlamydiales* order, which currently includes six different family-level lineages: the *Chlamydiaceae*, the *Parachlamydiaceae*, the *Waddliaceae*, the *Simkaniaceae*, the *Criblamydiaceae* and the *Rhabdochlamydiaceae* [1], [2]. All members of the *Chlamydiales* order exhibit a strict intracellular life cycle, with most undergoing a biphasic developmental cycle, beginning with an infectious, but metabolically inactive, elementary body (EB) entering its host cell by endocytosis and converting to a metabolically active reticulate body (RB) replicating by binary fission. At the end of the replication cycle, RBs redifferentiate into EBs, that are released by lysis of the host cell to initiate a new infection cycle [3].

The *Chlamydiaceae* family comprises well-known human and animal pathogens, of which several are implicated in adverse pregnancy outcomes and in respiratory tract infections in humans and in animals [4], [5], [6]. In recent years, attention has also turned to members of the *Parachlamydiaceae, Simkaniaceae* and *Waddliaceae* families, considered to be possible emerging human and animal pathogens. *Simkania negevensis* and *Parachlamydia acanthamoebae* are likely implicated in lower respiratory tract infections in humans [7], [8], [9]. Recent reports also indicate an association of *P. acanthamoebae* with abortion in ruminants [10], [11] and with human fetal loss [4], [12].

*W. chondrophila* is considered as an abortigenic agent in ruminants. It was isolated from aborted bovine fetuses on two separate occasions, once in USA and once in Germany [13], [14]. Furthermore, a serological study has demonstrated a clear association between anti-*Waddlia* antibodies and bovine abortion [15] and experimental infection of 2 fetuses with *W. chondrophila* led to the death of one within 2 weeks [15]. A pathogenic role of *W. chondrophila* in humans is supported by a serological study conducted on women having experienced sporadic or recurrent miscarriage, which demonstrated a strong association between *W. chondrophila* seropositivity and adverse pregnancy outcomes [16]. In addition, we recently implemented a real time quantitative PCR for the detection of this pathogen [17] and could report the presence of *W. chondrophila* DNA in placenta sample of a woman suffering from miscarriage [18]. *Waddlia chondrophila* was also detected in respiratory tract samples of patients with pneumonia and children with bronchiolitis [17], [19].

Given its obligate intracellular life cycle, *W. chondrophila* cannot be grown routinely on culture plates used to reveal pathogens and thus would remain undetected by conventional microbiological methods. In humans, the aetiology of miscarriage remains unknown in 50% of cases [16] and there is a clear need for improved methods to detect potential agents such as *W. chondrophila*. Until now, methods used to detect *W. chondrophila* infection have relied mainly on molecular techniques [17] or indirectly via seropositivity in micro-immunofluorescence or Western blots [16]. The latter are particularly time consuming and poorly applicable for screening large numbers of samples. Therefore, to further determine the pathogenic role of *W. chondrophila* in humans and in animals, there is an urgent need to identify immunogenic proteins and to establish a reliable ELISA test.

We recently reported the use of a combined genomic and immuno-proteomic approach, to identify immunogenic proteins of *Parachlamydia acanthamoebae* [20]. The same approach has also been used to identify immunoreactive proteins of *Bartonella quintana* and *Bartonella henselae* [21], [22], [23]. In this study, we combined an immunoproteomic method, i.e. a technique involving 2 dimensional gel electrophoresis followed by immunoblotting, with the complete genomic data of *W. chondrophila* that became recently available [24], to identify *W. chondrophila* immunogenic proteins and to establish the basis for a serological diagnostic test.

## Results

### 2D map of *W. chondrophila* immunoreactive proteins

**Figure 1:**
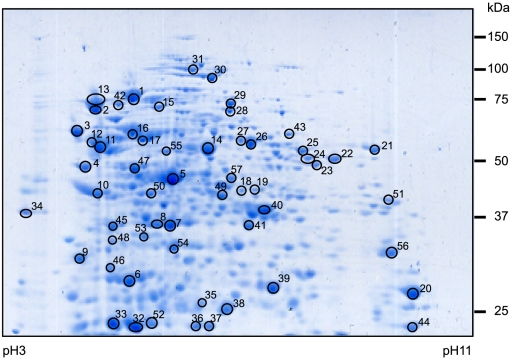
Coomassie blue-stained 2D map of the whole-protein extract of *W. chondrophila*. Proteins were transferred to nitrocellulose and probed with 13 *W. chondrophila* positive human sera and with the serum of a rabbit immunized with *W. chondrophila*. Immunoreactive proteins are numbered (1–57).

To identify immunoreactive proteins that could be used in a diagnostic test, elementary bodies of *W. chondrophila* were lysed and their proteins extracted and separated by 2 dimensional, polyacrylamide gel electrophoresis (2D gels). Bacterial proteins were subsequently either transferred onto nitrocellulose membranes or Coomassie-blue stained. To verify that the purification procedure retrieved only waddlial proteins, we also performed a 2D gel with proteins extracted from a mock control. Coomassie blue staining of this negative control revealed a total absence of proteins (data not shown). To detect immunoreactive proteins, blots were incubated with the serum of a rabbit immunized with *W. chondrophila* or with the sera of 13 patients previously tested positive for *W. chondrophila* by immunofluorescence [16], [25]. With the help of the Adobe Photoshop Software, the immunoreactive spots were matched with their corresponding proteins on the Coomassie blue-stained gel, which allowed the detection of 57 immunoreactive proteins (numbered 1 to 57 on Figure 1). The level of recognition of these 57 individual proteins by the 13 human *W. chondrophila* positive sera tested varied considerably, one being recognized by 92.31% of the sera (12/13) and others being detected by only 1 of the 13 tested sera (7.69%).

To be manually picked from the gel and accurately analyzed by mass spectrometry, a protein must be clearly stained by Coomassie blue and form a well defined spot. We selected 16 protein spots meeting these criteria as well as being recognized by at least 5 human *W. chondrophila* positive sera (of the 13 tested) and submitted them to mass spectrometry analysis. Spot number 24 was recognized by 4 sera, of which two very strongly, and was included in the analysis (Table 1).

**Table 1:** Reactivity of *W. chondrophila* positive sera towards 17 selected proteins.

Spot	Human *W. chondrophila* positive sera														
#	A	B	C	D	E	F	G	H	I	J	K	L	M	Frequency (%)	Rabbit serum
1	++	**−**	++	++	++	++	++	++	**−**	++	**−**	++	++	76.92	**−**
2	++	++	++	++	++	++	++	++	**−**	++	++	++	++	92.31	++
3	**−**	**−**	++	++	++	**−**	**−**	++	**−**	++	**−**	**−**	**−**	38.46	+
4	++	++	++	++	++	++	++	++	**−**	++	**−**	++	**−**	76.92	+
5	++	++	++	**−**	++	++	++	++	**−**	++	++	++	++	84.62	+
6	++	++	++	**−**	++	++	**−**	++	**−**	**−**	**−**	**−**	**−**	46.15	**−**
7	++	++	++	**−**	++	**−**	++	++	+	+	**−**	+	**−**	69.23	**−**
11	++	++	++	++	++	++	++	++	++	**−**	++	++	**−**	84.62	++
13	**−**	+	+	+	+	+	+	+	**−**	+	+	+	+	84.62	**−**
16	**−**	+	**−**	+	+	+	+	+	+	**−**	**−**	+	+	69.23	**−**
17	**−**	++	**−**	++	++	**−**	++	++	**−**	**−**	**−**	**−**	++	46.15	**−**
20	**−**	**−**	**−**	**−**	+	+	+	+	**−**	**−**	**−**	**−**	+	38.46	**−**
21	**−**	+	+	+	**−**	+	+	**−**	**−**	**−**	**−**	+	**−**	46.15	**−**
22	+	+	+	+	+	+	+	**−**	**−**	**−**	**−**	**−**	+	61.54	**−**
23	+	+	**−**	++	++	+	+	**−**	**−**	**−**	**−**	**−**	+	53.85	**−**
24	+	**−**	+	++	++	**−**	**−**	**−**	**−**	**−**	**−**	**−**	**−**	30.77	**−**
56	**−**	++	++	**−**	++	++	++	**−**	**−**	**−**	**−**	**−**	**−**	38.46	**−**

The reactivity of 17 proteins of *W. chondrophila* selected for mass spectrometry analysis towards 13 human and 1 rabbit sera was investigated by western blot. The signal intensities are presented semi-quantitatively: (−) negative, (+) positive, (++) strongly positive.

### Identification of immunogenic proteins

MALDI TOF MS analysis of 17 individual spots corresponding to immunoreactive proteins resulted in the identification of 13 different proteins, mainly already known chlamydial antigens and proteins described as immunogenic in other bacterial species [22], [26] (Table 2). More interestingly, this immunoproteomic method allowed the detection of two hypothetical proteins, spot #3 (Wim3) and spots #4 and #7 (Wim4). Wim3 has no homology with any known protein and is therefore a very promising candidate for the development of a *W. chondrophila* specific ELISA test. Wim4 displays no sequence homology with proteins of *C. trachomatis* and of other members of the *Chlamydiaceae* family, but partial homologs are found in the genomes of two members of the *Parachlamydiaceae* family, *P. amoebophila* (pc1399, 34% identity) and *P. acanthamoebae* (pah_c029o043, 41% identity) as well as in the draft genomes of two members of the *Criblamydiaceae* family, *Estrella lausannensis* (35% identity) and *Criblamydia sequanensis* (36% identity) (C. Bertelli *et al*., unpublished results). The sequence homologies are mainly restricted to the C-terminal region (last 100 amino acid residues) of these related proteins. Moreover, the *P. acanthamoebae* homolog of Wim4 has been previously identified as an immunogenic protein [20]. Interestingly, the *W. chondrophila* gene wcw_1618, encoding hypothetical protein #4 (Wim4), is located in one of the four type III secretion system (T3SS) genetic clusters present on the bacterial chromosome [24].

**Table 2:** Identification of 13 different immunogenic proteins of *W. chondrophila* and amino acid sequence identity with 2 related organisms.

Spot number	ORF	Accession number^2^	Protein identification	Amino acid sequences identity (%) with	
(protein name)^1^				*P. acanthamoebae*	*C. trachomatis* 434/Bu
1 (Wim1)	wcw_0306	GENE ID: 9277285 fusA	Elongation factor G	78	74
2 (Wim2)	wcw_1638	GENE ID: 9278617 dnaK	Chaperone protein DnaK	77	71
3 (Wim3)	wcw_1327	GENE ID: 9278306 wcw_1327	Hypothetical protein	No homolog	No homolog
4 (Wim4)	wcw_1618	GENE ID: 9278597 wcw_1618	Hypothetical protein	41	No homolog
5 (Wim5)	wcw_0584	GENE ID: 9277563 tuf	Elongation factor Tu	79	74
6 (Wim6)	wcw_1934	GENE ID: 9278912 tsf	Elongation factor Ts	65	41
7 (Wim4)	wcw_1618	GENE ID: 9278597 wcw_1618	Hypothetical protein	41	No homolog
11 (Wim11)	wcw_0859	GENE ID: 9277838 nusA	Transcription elongation protein NusA	75	61
13 (Wim2)	wcw_1638	GENE ID: 9278617 dnaK	Chaperone protein DnaK	77	71
16 (Wim16)	wcw_1343	GENE ID: 9278322 groEL1	Chaperonin GroEL	81	75
20 (Wim20)	wcw_0589	GENE ID: 9277568 rplA	50S ribosomal protein L1	70	58
21 (Wim21)	wcw_0972	GENE ID: 9277951 pepA	Leucyl aminopeptidase	No homolog	46
22 (Wim22)	wcw_1647	GENE ID: 9278626 rho	Transcription termination factor rho	85	79
23 (Wim23)	wcw_0999	GENE ID: 9277978 lpdA2	2-oxoglutarate dehydrogenase E3 component	62	38
24 (Wim22)	wcw_1647	GENE ID: 9278626 rho	Transcription termination factor rho	85	79

1 Wim: *Waddlia* immunogenic protein,

2 Genome accession number: GenBank CP001928.

Identification and amino acid sequence identities of each immunogenic protein identified by mass spectrometry are provided according to BLASTP results against a non-redundant database.

### Western blot and ELISA with rabbit sera

Most of the 13 identified immunogenic proteins described above have highly conserved homologs in the genomes of other *Chlamydiales* bacteria (Table 2) and are thus likely to generate cross-reactions if used in an ELISA test. For this reason, only the two hypothetical proteins Wim3 and Wim4 were further considered for the development of a *W. chondrophila*-specific serological diagnostic tool. *In silico* analysis of their amino acid sequences revealed the absence of signal peptides and transmembrane domains [27], [28], [29]. Both proteins were expressed in *E. coli* and purified via a C-terminal His tag. The purified recombinant proteins were detected by Western blot with a rabbit anti-*Waddlia* serum but not with the corresponding pre-immune serum (data not shown).

Both Wim3 and Wim4 were detected by sera of two rabbits immunized with *W. chondrophila* in a direct ELISA assay. Serum of rabbit #2 was reactive against the two proteins down to a dilution of 1/256 while serum of rabbit #1 remained reactive at a dilution of 1/4096. Only background reactions were observed with the corresponding pre-immune sera (data not shown). This demonstrated that Wim3 and Wim4 could be detected by the sera from animals immunized with the whole bacterium, supporting their potential use in a serological diagnostic test.

### ELISA with human sera

**Figure 2:**
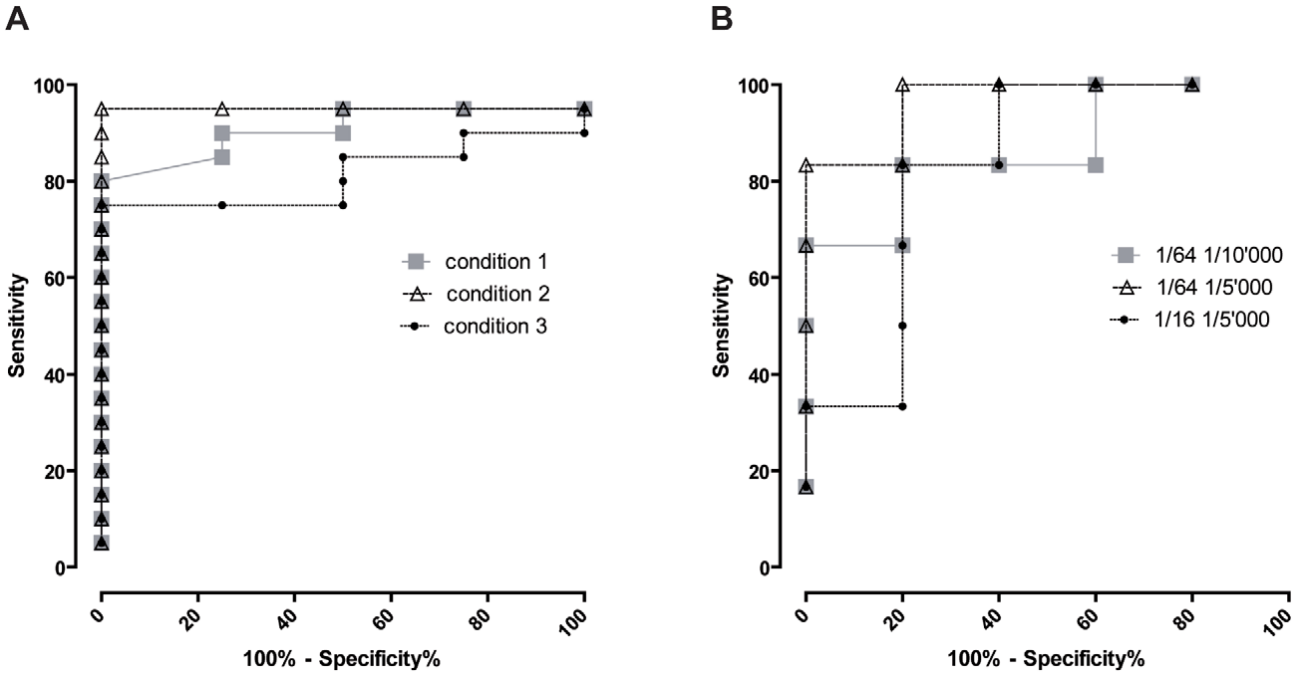
Optimization of ELISA conditions. A. ROC curves obtained using Wim 3 protein as antigen. Combination of buffers used for saturation and dilution of antibodies as well as for washing steps were tested with sera positive and negative for *W. chondrophila*. See Material and Methods for further description of the assay conditions. B. ROC curves obtained with the Wim3 assay using condition 2 and various dilution of primary and secondary antibodies.

**Figure 3:**
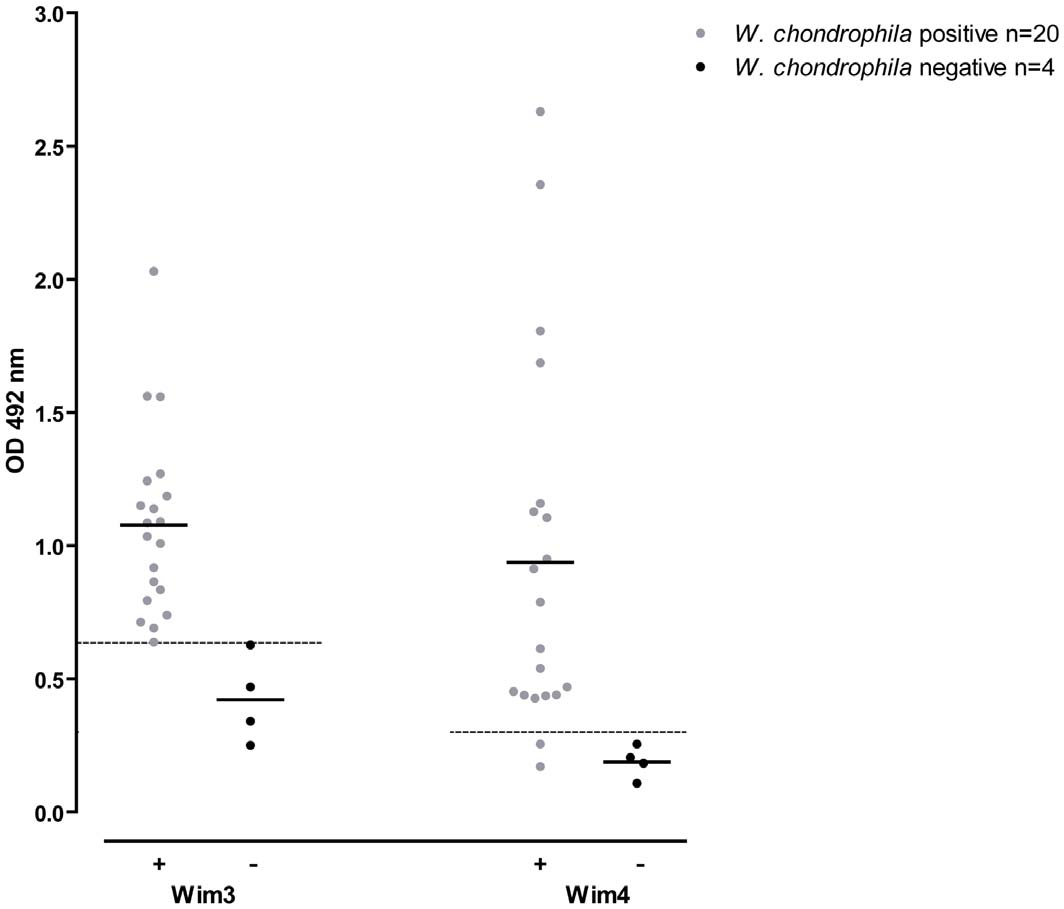
Analysis of 24 human sera by ELISA. 96-well ELISA microplates were coated either with recombinant protein Wim3 or recombinant protein Wim4. Human sera were previously characterized by MIF and are represented as follows: grey circles: *W. chondrophila* positive (n = 20); black circles: *W. chondrophila* negative (n = 4). The bar indicates the mean value for each group. Threshold of positivity: 0.63 for Wim3 and 0.30 for Wim4. Results are the mean of three independent experiments. Thresholds were determined by ROC curves analyses (GraphPadPrism).

To evaluate the sensitivity and specificity of Wim3 and Wim4 proteins in a *W. chondrophila* ELISA, we utilized 24 human sera from a previous study [16] and we first determined their positivity or negativity towards *W. chondrophila* using a micro-immunofluorescence assay (MIF) as gold standard. The sera reactivity was then assessed by direct ELISA using Wim3 and Wim4 as antigens. In order to maximize the signal∶noise ratio of these assays, we performed optimization experiments in which various primary and secondary antibody dilutions as well as different saturation and washing conditions were compared. The optimal assay conditions were determined using ROC analyses (Figure 2). In these optimized conditions, the results obtained with 20 *W. chondrophila* positive and 4 *W. chondrophila* negative human sera indicated that both Wim3 and Wim4 assays are able to distinguish the two groups of sera (Figure 3). Considering a threshold value of 0.63, the sensitivity was 100% for the Wim3 assay and, with a threshold value of 0.3, 90% for the Wim4 assay. In addition, sera negative for *W. chondrophila* and for other members of the *Chlamydiales* order were not detected neither in the Wim3 nor in the Wim4 assay (specificity of 100% for both assays, see Figure 3).

## Discussion

*W. chondrophila* is now considered as an abortigenic agent in ruminants [13], [14], [15] and as a possible cause of adverse pregnancy outcome in humans [16], [18]. Moreover, bacterial DNA has been detected in respiratory tract samples from patients with pneumonia and from children with bronchiolitis [17], [19]. Recent publications from our group documented the intracellular growth of *W. chondrophila* in human macrophages, endometrial cells and pneumocytes [30], [31], [32], which further underlines the pathogenic potential of this organism for humans. At present, serological investigation to diagnose *W. chondrophila* infection relies on a tedious and time-consuming micro-immunofluorescence (MIF) test [16], [25]. Given its pathogenic potential, it is crucial to develop a reliable, sensitive and specific ELISA test for assessing the prevalence of this organism in the human population in large sero-epidemiological studies.

In order to identify immunogenic proteins of *W. chondrophila* that would be family-specific, we established a combined immunoproteomic and comparative genomics approach utilizing newly available genomic data [24]. This led to the identification of *Waddlia* proteins, homologs of known chlamydial immunogenic proteins such as chaperonin GroEL (Hsp60), chaperone protein DnaK (Hsp70), elongation factor Tu and leucyl aminopeptidase [33], [34], [35] and of proteins, such as elongation factor G, elongation factor Ts, transcription termination factor rho and 2-oxoglutarate dehydrogenase E3 component which have been described as immunogenic in other bacterial species [22], [26]. This congruence between our results and those reported by other scientists using other approaches supports the relevance of our immunoproteomic method to successfully identify immunogenic proteins. Furthermore, all proteins identified by mass spectrometry were of waddlial origin, a result that reveals the purity of the bacterial preparation, further confirmed by the complete negativity of the 2D gel obtained with the mock control.

Four of the 13 identified proteins (chaperonin GroEL, chaperone protein DnaK, elongation factor Tu and elongation factor Ts) were also described as immunogenic in a similar work conducted on *P. acanthamoebae* [20]. In addition, *W. chondrophila* hypothetical protein #4 (Wim4), also identified in this work, is homolog to a *P. acanthamoebae* hypothetical protein (pah_c029o043), which was previously shown to be immunogenic [20].

Commercially available ELISA tests for the diagnosis of chlamydial infections are usually based on recombinant peptides of the major outer membrane protein (MOMP) or of the 60 kDa cysteine-rich outer membrane protein (OmcB), two major antigens of the organisms belonging to the *Chlamydiaceae* family [33], [36], [37]. Although, no homolog to MOMP could be identified in the *P. acanthamoebae* or in the *P. amoebophila* genomes, a putative cysteine-rich MOMP-family is present in the *W. chondrophila* genome [20], [24], [38]. However, no protein of this family was identified in the present work, most probably because we had, on purpose, chosen extraction and isolation conditions which should leave the putative waddlial disulphide linked matrix as an insoluble complex. Since our primary aim was to identify unique immunogenic proteins which we could express as fusion-proteins in bacteria for use in a high-throughput ELISA and since disulphide-rich fusion proteins are notoriously difficult to prepare in this manner, we had decided to leave this aspect for a later date. This strategy has the added advantage of easing the preparation of clean 2D gels for proteomic analysis and immunoblotting.

In addition, several other proteins, such as ribosomal proteins S1 and L7/L12, chlamydial protease-like activity factor (CPAF), translocated actin-recruiting protein (TARP) and polymorphic membrane protein D (PmpD), which are well-described antigens of *Chlamydiaceae* [34], [35], [39], [40] were also not detected in our study on *W. chondrophila*. Since the corresponding genes are indeed present in the *Waddlia* genome, these proteins are likely among those that were not chosen for analysis by mass spectrometry, being either weakly immunogenic or poorly expressed in elementary bodies. Alternatively, large proteins such as the waddlial putative Pmp could be under represented due to the 2D gel limitations for large proteins.

Despite these limitations, our strategy proved to be successful, with 2 novel proteins (Wim3 and Wim4) selected (from the 13 identified immunogenic proteins) as promising candidates for the development of a *Waddlia*-specific ELISA since they display little or no sequence homology with proteins of other members of the *Chlamydiales* order. Indeed, one recurrent problem of previous serological studies in the chlamydial field was the possible cross reactivity of highly conserved proteins, such as Hsp60, with different bacteria of the same phylogenic order. This limitation was overcome in the present study by the use of a protein not encoded in the genome of any *Chlamydiales* bacteria sequenced today [20], [41], [42]. In Western blot as well as in ELISA, both recombinant proteins Wim3 and Wim4 reacted with the sera of rabbits immunized with *W. chondrophila* and not with the corresponding pre-immune sera thus confirming the potential of these two proteins for use in a diagnostic serological test. Wim3 and Wim4 were used in ELISA to assess the reactivity of human sera previously tested by a micro-immunofluorescence (MIF) technique (considered as the gold standard for serological diagnosis) towards *W. chondrophila*. The sensitivity of the ELISA was 100% for the assay with Wim3 and 90% for the assay with Wim4, using cut off values of 0.63 for the Wim3 assay and of 0.3 for the Wim4 assay, respectively. When considering *W. chondrophila* negative sera, the specificity of both assays was 100% with the defined cut off values. Thus, the results obtained with Wim3 and Wim4 ELISA were congruent with those obtained by MIF in 100% and 92% of the cases respectively, despite the fact that the antibody response measured is directed against one specific protein in the ELISA and against the whole bacterium in the MIF tests.

In conclusion, by combining genomic and proteomic approaches, this work allowed the identification of an immunogenic protein set for *W. chondrophila*, of which a number are highly conserved homologs of well-known chlamydial antigens. In addition, we also reported the immunoreactivity of two novel proteins of unknown function, one (Wim3) being specific to *W. chondrophila.* Preliminary experiments indicated that a direct ELISA test using Wim3 as antigen is able to discriminate between *Waddlia* positive and *Waddlia* negative sera as efficiently as micro-immunofluorescence and could be used as an alternative to screen large numbers of samples. Further work is now needed to precisely establish the specificity of this assay, in particular towards other members of the *Chlamydiales* order.

## Methods

### Human sera

Human sera were described in previous studies [16], [25]. The clinical studies were approved by the local ethical committees. All sera were tested with an in-house micro- immunmofluorescence method (see below) for reactivity against *W. chondrophila.* In addition, negative sera were also tested for reactivity against other members of the *Chlamydiales* order (*P. acanthamoebae, E. lausannensis, C. sequanensis, S. negevensis, N. hartmannellae, C. trachomatis, C. pneumoniae* and *C. psittaci*) [16].

### Sera from immunized rabbits

Rabbit anti-*W. chondrophila* sera were obtained by vaccination with *W. chondrophila* strain ATCC VR-1470 grown in amoebae (see below). Briefly, rabbits were inoculated at days 0, 14, 28 and 56 with 500 µl of PBS containing 5×10^8^ heat-inactivated *W. chondrophila*. Animals were bled prior to immunization (pre-immune sera) and at day 90.

### Culture and purification of *Waddlia chondrophila*

*W. chondrophila* strain ATCC VR-1470 was grown at 32°C within *Acanthamoeba castellanii* strain ATCC 30010 in 25 cm^2^ or 75 cm^2^ cell culture flasks (Corning, New York, USA) with 10 or 30 ml of peptone-yeast extract-glucose broth and purified using a sucrose barrier and a gastrografin gradient, as previously described [6], [43]. The same purification procedure was also performed on non-infected *A. castellanii* (mock control). The purified bacteria were conserved in PBS and 20% glycerol at −80°C.

### Crude extract sample preparation and 2D gel electrophoresis

Bacterial cells resuspended in PBS were washed in 10 mM Tris, 5 mM Magnesium acetate, pH 8.0 and then lysed by 5 cycles of short-pulse sonication in lysis buffer (30 mM Tris, 7 M urea, 2 M thiourea, 4% CHAPS, pH 8.5). Proteins were recovered by centrifugation at 6'000 g and their concentration determined using a Bradford assay (Quick Start™ Bradford Protein Assay, Biorad, Hercules, USA).

Two dimensional gel electrophoresis was performed as described by Centeno et al. [44] using approximately 150 µg (mini gels) or 600 µg (midi-gels) of total elementary bodies proteins. Proteins were visualized by Coomassie Blue staining or transferred to nitrocellulose for subsequent immunoblot analyses.

### Immunoblot analysis

Nitrocellulose membranes corresponding to 2D gels were blocked by 2 hours incubation with 5% non-fat dry-milk in Tris-buffered saline with 0.05% Tween 20 (TBS), washed 3 times with TBS, 0.5% milk and incubated overnight at 4°C with sera diluted in TBS, 0.5% milk. Membranes were probed either with human sera (dilution 1/8) or with sera of immunized rabbits (dilution 1/25). After 3 subsequent washes with TBS, 0.5% milk, membranes were incubated with horseradish peroxidase-conjugated goat anti-human IgG (Chemicon, Temecula, CA, 1∶500) or horseradish peroxidase-conjugated goat anti-rabbit IgG (Cell Signaling, Allschwill, Switzerland, 1∶1000). Finally, membranes were washed 3 times with TBS and immunoreactive spots were detected either with a chemiluminescence-based kit (LiteAblot™, Euroclone SpA, Pero, Italy) or with 4-chloro-1-naphtol (Sigma-Aldrich, St. Louis, MO, USA).

The same protocol was used for 1D western blots on Wim3 and Wim4 using sera of rabbits immunized with *W. chondrophila* or preimmune sera at dilution 1/100.

### Mass spectrometry

For identification of proteins, Coomassie Blue stained spots were excised from 2D gels and transferred to special 96-well plates (Perkin Elmer Life Sciences). In-gel proteolytic cleavage with sequencing-grade trypsin (Promega, Madison, WI, USA) was performed in the automated workstation Investigator ProGest (Perkin Elmer Life Sciences) according to the protocol of Shevchenko *et al.* [45]. Digests were evaporated to dryness and resuspended in 3 µl alpha-cyano-hydroxycinnamic acid matrix (5 mg/ml in 60% (v∶v) acetonitrile∶water), of which 0.7 µl were deposed in duplicate on a target plate.

MALDI-MS-MS analysis was performed on a 4700 Proteomics Analyser (Applied Biosystems, Framingham, MA, USA). After MALDI-TOF MS analysis, internal calibration on trypsin autolysis peaks and subtraction of matrix peaks, the 10 most intense ion signals were selected for MS/MS analysis. Non-interpreted peptide tandem mass spectra were used for direct interrogation of *W. chondrophila* open reading frames using Mascot 2.0 (http://www.matrixscience.com). The mass tolerance for database searches was 50 ppm. With the parameters used, the threshold for statistical significance (p<0.05) corresponded to a MASCOT score of 17, but only scores greater than 30 were considered. Proteins scoring above 80 were automatically considered as valid, while all protein scoring between 30 and 80 were manually validated. Validation included examination of the peptide mass error of individual peptide matches. When proteins could not be identified by MALDI-TOF MS analysis, spots were reanalyzed by LC-MS followed by the same identification procedure as described above.

### Cloning, expression and purification of immunogenic proteins

The ORFs corresponding to the immunogenic proteins #3 (Wim3);(ORF wcw_1327) and #4 (Wim4);(ORF wcw_1618) were amplified by PCR and cloned into the pET28 vector (Novagen EMD Chemicals Inc, San Diego, USA). This vector system allows to express the protein of interest fused with a C-terminal 6-His tail. Protein expression was induced during 2.5 hours with 1 mM isopropyl-β-D-thiogalactopyranoside (IPTG, Qbiogen, Basel, Switzerland). The expressed proteins were purified under non denaturating conditions as previously described [20] and concentrated using a Macrosep^R^ 10K (Pall Corporation, Port Washington, USA). Final protein concentrations were determined using the Bradford Quick Start™ assay (Biorad).

### Micro-immunofluorescence assay (MIF)

Purified bacteria were inactivated by incubation with 0.3% formalin during 1 hour at room temperature before being spotted on MIF glass slides (12 well diagnostic slides, Menzel, Braunschweig, Germany) previously washed with 70% ethanol. Slides were pre-incubated with PBS 1% BSA during 1.5 hours at room temperature in a humidified chamber, then washed for 5 minutes with PBS 0.03% Tween 20, 5 minutes with PBS and 5 minutes with distilled water. Human sera to be tested were diluted 1/32 in PBS 1% BSA and added to the antigens as a 25 µl drop. After one hour incubation at room temperature in a humidified chamber, slides were washed as before. Slides were then incubated one hour at room temperature with a 1/400 dilution of goat anti human IgH-fluorescein conjugated (Fluoline H, Biomerieux, France) and 150 ng/ml DAPI (dilactate, Molecular Probes, OR, USA) in PBS 1% BSA. After 3 washing steps as before, slides were mounted in Mowiol (Sigma-Aldrich, MO, USA). Positive, negative or doubtful immunofluorescence signals were determined by two independent investigators using an epifluorescent microscope (Axioplan 2, Zeiss, Feldbach, Switzerland) at a magnification of 100×.

### ELISA

#### Optimization of signal∶noise ratio

To optimize the assay with human sera, different saturation and washing conditions were compared. Buffers used to block and to dilute primary and secondary antibodies were:

condition 1: PBS, 0.1% Tween 20, 1% non-fat dry milk

condition 2: 20 mM Tris-HCl pH 7.5, 0.11 M NaCl, 0.1% Tween-20, 10% FCS, 1% BSA, 1% casein, 1% PEG

condition 3: same as condition 2 but 0.5 M NaCl

condition 4: same as condition 2 but 1 M NaCl

condition 5: same as condition 2 but 1.5 M NaCl

For each condition, a related washing buffer was used:

condition 1: PBS, 0.1% Tween-20

condition 2: 20 mM Tris-HCl pH 7.5, 0.11 M NaCl, 0.1% Tween-20

condition 3: same as condition 2 but 0.5 M NaCl

condition 4: same as condition 2 but 1 M NaCl

condition 5: same as condition 2 but 1.5 M NaCl.

In the optimization phase, human sera were diluted 1/16, 1/32, 1/64 and 1/128. Secondary antibody (horseradish peroxidase-conjugated goat anti-human IgH (Chemicon, Temecula, CA)) was diluted 1/5'000, 1/10'000, 1/25'000 and 1/50'000. The enzymatic reaction with O-phenylenediamine dihydrochloride (OPD) as substrate was stopped with 3M H_2_SO_4_ after 5, 15 or 45 minutes and the optical density was read at 492 nm using an ELISA reader (Multiskan Ascent, Thermo Scientific, Waltham, USA). ROC analyses of the ELISA outcome were performed with GraphPad Prism version 5.04 for Windows (GraphPad Software, San Diego, CA, USA).

#### Wim3 assay

96-well microplates were coated during 12 hours at 4°C with 100 ng/well of purified protein in carbonate/bicarbonate buffer pH 9.6. Saturation as well as primary and secondary antibody dilutions were performed in 20 mM Tris-HCl pH 7.5, 0.11 M NaCl, 0.1% Tween-20, 10% FCS, 1% BSA, 1% casein, 1% PEG. The saturation step of 1 hour at 37°C was followed by one washing step with 20 mM Tris-HCl pH 7.5, 0.11 M NaCl, 0.1% Tween-20 and a 2 hours incubation at 37°C with primary antibodies diluted 1/64. After 5 washing steps, the plates were incubated during 1 hour at 37°C with a 1/5000 dilution of horseradish peroxidase-conjugated goat anti-human IgH. After 5 washing steps, substrate (1 mg/ml O-phenylenediamine dihydrochloride in citrate buffer) was added and the enzymatic reaction was stopped with 3M H_2_SO_4_ after 5 minutes. The optical density was read at 492 nm.

#### Wim4 assay

Same protocol as for Wim3 assay except that saturation and primary/secondary antibody dilutions were performed in PBS, 0.1% Tween-20, 1% milk and that washing steps were performed using PBS, 0.1% Tween-20. The enzymatic reaction was stopped after 15 minutes.

When testing rabbit sera, ELISA were performed as described elsewhere [20].
